# Risk factors and methods in suicides of elderly patients connected to mental health services from 1999–2024

**DOI:** 10.3389/fpsyt.2024.1425371

**Published:** 2024-06-17

**Authors:** Eric C. Chan, Kim Conlon, Lisa Gagnon

**Affiliations:** ^1^ Department of Psychiatry, University of Calgary, Calgary, AB, Canada; ^2^ Mathison Centre for Mental Health Research and Education, University of Calgary, Calgary, AB, Canada; ^3^ Alberta Health Services, Calgary, AB, Canada

**Keywords:** suicide, depression, elderly, psychiatric services, suicide risk factors, suicide methods, psychiatric hospitalization

## Abstract

**Introduction:**

Suicide prevention is an important aspect of psychiatric care, with older men being a population identified at especially high suicide risk and a recent increase in suicides among older women.

**Methods:**

Using data collected by the region’s quality assurance team, we examined all suicide deaths occurring between March 1999 and February 2024 in patients aged 60 years or older who were connected to the region’s Addiction and Mental Health Program at the time of death. Data were analyzed to describe which factors were most commonly identified in suicides in older adults receiving mental healthcare. We also compared male and female cases to determine whether certain factors were more commonly observed in one gender.

**Results:**

We identified 48 cases of suicide occurring in patients aged 60 or over. 60% of suicides occurred in males. Overdose and hanging were the most common suicide methods used, and all suicides occurring on inpatient units occurred via hanging. Depression was the most common diagnosis, and was diagnosed more frequently in suicides of female older adults. A greater proportion of suicides in older women were associated with previous history of suicide attempts.

**Discussion:**

Our findings support many current best practices for suicide prevention in psychiatric care, including minimizing ligatures and anchor points on inpatient settings, assessing for and limiting access to means in individuals at-risk, and assessing suicide risk in hospitalized patients prior to passes and discharge. Recognition and treatment of depression remain important aspects in the treatment of older adults to prevent suicide.

## 1 Introduction

Suicide prevention is an important aspect of the psychiatric care of the elderly, with older men in particular being a group often identified as at especially high risk. In 2019, males aged 85 to 89 years were the sex and age group with the highest rate of suicide at 27.7 per 100,000 population and males aged 90 years and older had the highest rate in 2020 at 20.3 per 100,000 (1). Furthermore, among women in the U.S., the only significant increase in suicides between 2020 and 2021 occurred in the population aged 75 and over (2).

Psychiatric disorders, non-psychiatric medical illnesses, social isolation, substance use, and recent loss/adverse events have been identified as risk factors of suicide in the general older age population (3, 4). Depression is the most common psychiatric diagnosis occurring in older adults who died from suicide, being especially common in women receiving mental healthcare at the time of death (5). The presence of non-psychiatric medical illness is also common in suicides occurring in older adults, in particular among older men (4, 6).

While many older adult suicides occur in individuals with no psychiatric diagnosis or contact with mental health services, involvement with mental health services is still common, occurring in more than 30% of suicides in older women and more than 20% of suicides in older men (4, 5). Of note, the presence of non-psychiatric medical illness is more common in older adult suicides without known mental illness (4). Likewise, the use of firearms is less common in cases without depression or known mental illness (4, 5). Contact with mental health services is an important opportunity to take steps to assess for and address factors contributing to suicide risk. Consequently, an understanding of the unique factors associated with suicide in older adults in the mental healthcare system is important in improving suicide prevention efforts.

To describe the factors associated with suicide in older adults receiving mental healthcare services, we analyzed data collected by the quality assurance team in Calgary, Canada on suicides occurring in older adult patients connected due the region’s Addiction and Mental Health Program at the time of death. Our aim is to identify which factors are most common, to inform our understanding of interventions that can be developed within the mental healthcare system, and to identify differences that may exist between genders in this setting.

## 2 Methods

This study examines suicide deaths occurring in elderly patients receiving psychiatric care between March 1999 (when the quality assurance team began collecting these data) and February 2024. We examined all deaths from suicide occurring in patients aged 60 years or older who were receiving care from services within the municipal region’s Addiction and Mental Health Program at the time of death. Cases include patients who died from suicide:

- While followed by an outpatient addiction and mental health program.
- While on a waitlist for an outpatient addiction and mental health program.
- Seen by a psychiatric emergency services team in the emergency department in the 72 hours prior to death.
- While admitted to a psychiatric unit.
- In the month following a psychiatric admission.
- After being seen by the psychiatric consultation liaison team while admitted to another medical service within the 30 days prior to death.

The quality assurance team was alerted to possible suicides by clinical services involved in the patient’s care (both psychiatric and non-psychiatric services such as intensive care), family members of the individual, and/or the medical examiner’s office.

Data on the date of death, clinical service most recently involved in the patient’s care, gender, age, ethnicity, marital status, employment status, psychiatric diagnosis, history of substance use, history of suicide attempts and self-harm, presence of social isolation, and events preceding death from suicide were collected by the quality assurance team. These factors were chosen given their previous association with suicide risk in various studies, including studies of suicide in older adults (3, 4). These data were obtained through various sources, including review of discharge summaries, psychiatric assessments, progress notes, and notes obtained from collateral sources by the treatment team that had been managing the patient’s care. The primary diagnosis and all other diagnoses were identified from the most recent comprehensive psychiatric assessment or discharge summary. Information on suicide method was obtained through the medical examiner’s report or information provided to the quality assurance team by the individual’s loved one.

Data was analyzed using IBM SPSS version 29.0.0.0 (241) to describe the demographic characteristics of cases identified, as well as the most recent care setting, suicide method used and presence/absence of adverse life events identified in the period preceding death. To determine whether an association existed between gender and marital status, employment status, suicide method, presence of adverse events in the preceding 6 months, psychiatric diagnosis, history of substance use disorder and history of self-harm/suicide attempt, Fisher’s exact test. This test was selected as it can be used for small sample sizes such as the sample used in this study.

## 3 Results

In total, we identified 48 cases of suicide occurring in patients aged 60 or over. No statistically significant association between gender and variables listed above was identified when performing Fisher’s Exact Test, however the small sample size limits our ability to interpret this. Some patterns were observed in which there was a greater than 10% difference in proportion within each gender with certain characteristics and these are reported to inform future studies. Detailed results can be found in **Supplementary Appendix 1**.

### 3.1 Demographic characteristics

Demographic characteristics of suicide deaths are described in **Table 1**. 29/48 (60%) of suicide deaths occurred in males. The majority (34/48; 71%) of suicide deaths occurred in patients aged 60 – 69. Notably, all five suicides occurring in patients 80 or older were male. Likewise, almost all (44/48; 91%) of patients were Caucasian, with four additional cases identified as Hispanic, Southeast Asian, East Asian, or Jewish.

**Table 1:** Demographic factors.

	n (%)
Total	48
Gender	
Female Male	19 (40%) 29 (60%)
Age at time of death (years)	
60 - 69 70 – 79 80 – 89 90 +	34 (71%) 9 (19%) 3 (6%) 2 (4%)
Ethnicity	
Caucasian Hispanic Southeast Asian East Asian Jewish	44 (91.7%) 1 (2.1%) 1 (2.1%) 1 (2.1%) 1 (2.1%)
Marital status	
Married Single Divorced Separated Widowed	22 (46%) 7 (15%) 10 (21%) 3 (6%) 6 (13%)
Employment status	
Unemployed Unemployed with disability income Partially employed with disability income Employed Retired Unknown	13 (27%) 4 (8%) 1 (2%) 3 (6%) 24 (50%) 3 (6%)

Slightly less than half (22/48; 46%) of elderly patients that died from suicide were married, and roughly one quarter (13/48; 27%) were divorced or separated at the time of death. A greater proportion of females were married at time of death compared to males (Male: 38%; Female 58%). Half (24/48; 50%) of elderly patients that died from suicide were retired, and many were unemployed (13/48; 27%), unemployed with disability income (4/48; 8%) or partially employed with disability income (1/48; 2%) at the time of death.

### 3.2 Most recent treatment setting and suicide method

Data on the most recent treatment setting and suicide method are detailed in **Table 2**. Suicide deaths occurred in all treatment settings examined, with more than half (27/48; 56%) of suicide deaths occurring in patients receiving care in an outpatient psychiatric setting. Half of all suicide deaths associated with an inpatient psychiatric admission (admitted patient or recently discharged) occurred while the patient was on a weekend or overnight pass while admitted (6/12; 50%) and another quarter occurred in patients recently discharged.

**Table 2:** Treatment setting and suicide method.

	n (%)
Total	48
Most recent treatment setting	
Outpatient psychiatric management On waitlist for outpatient treatment Psychiatric inpatient Discharged by treatment team Discharged against medical advice On psychiatric unit Off-unit while admitted On weekend/overnight pass during admission Seen by consultation liaison team Assessed and discharged from ED Continuing care facility Addiction program	27 (56%) 1 (2%) 2 (4%) 1 (2%) 2 (4%) 1 (2%) 6 (13%) 1 (2%) 2 (4%) 1 (2%) 4 (8%)
Method	
Unknown Laceration Drowning Overdose Firearm Hanging Carbon Monoxide Fall from Height Asphyxiation	7 (15%) 1 (2%) 4 (8%) 15 (31%) 2 (4%) 15 (31%) 1 (2%) 2 (4%) 1 (2%)

Overdose and hanging were the most common suicide methods used in identified cases, with each method used in 15/48 (31%) of deaths. In 7/48 (15%) of cases, the suicide method was not identified in the quality assurance team record. Of note, hanging was the suicide method in both deaths that occurred on an inpatient psychiatric unit and in the suicide death occurring in a continuing care facility. While hanging and overdose were the two most common suicide methods for both genders, a greater proportion of males died from hanging (Male: 35%; Female: 26%) and a greater proportion of females died from overdose (Male: 24%; Female: 42%).

### 3.3 Adverse life events prior to death

45 cases had data recorded on whether adverse life events occurred in the 6 months preceding death, with details described in **Table 3**. Of the 45 cases with recorded data, 32 (71%) had adverse life events identified in the 6 months preceding death. Many patients had more than one adverse life event in the 6 months prior to death, with a newly identified medical issue (10/45; 22%) being the most common adverse event identified. Loss of an important social support was also frequently identified, and this may have occurred through severe illness or death of a loved one (6/45; 13%) or for other reasons (5/45; 11%) (ex. adult children travelling or moving away, physical impairments preventing patient from participating in previous social activities, decreased contact with friends). While financial stress was only identified in 3 out of 45 cases (7%), loss of housing was identified as a stressor for 6 out of the 45 cases (13%). These data are summarized in **Table 3**.

**Table 3:** Adverse life events in 6 months preceding death.

	n (%)
Total	45
Type of adverse life event in preceding 6 months	
Any adverse life event in preceding 6 months New medical issue Illness or death in loved one Loss of important social support for other reasons Loss of housing Financial stress Social conflict	32 (71%) 10 (22%) 6 (13%) 5 (11%) 6 (13%) 3 (7%) 1 (2%)

When examining whether social isolation or medical illness were present regardless of the time of onset, social isolation was identified in 30 out of 46 cases (65%) and any history of medical illness was identified in 34 out of 48 cases (71%). The proportion of cases with adverse life events in the preceding six months were similar between men and women. A slightly higher proportion of males had a history of medical illness (Male: 76%; Female: 63%).

### 3.4 Psychiatric history

**Table 4** summarizes the frequencies of diagnosis in elderly patients that died from suicide. Each record had a single primary diagnosis but may have had multiple additional diagnoses listed. The number of cases with a history of substance use disorder or previous self-harm/suicide attempt is also included in **Table 4**.

**Table 4:** Psychiatric history.

	n (%)
Total	48
Primary diagnosis	
Depression Bipolar disorder Adjustment disorder Schizophrenia or other psychotic disorder Anxiety Substance-related disorder Grief Cluster B personality disorder Dementia Somatoform disorder	23 (48%) 3 (6%) 3 (6%) 4 (9%) 5 (11%) 5 (11%) 2 (4%) 1 (2%) 1 (2%) 1 (2%)
All diagnoses	
Depression Bipolar disorder Adjustment disorder Schizophrenia or other psychotic disorder Anxiety Substance-related disorder Grief Cluster B personality disorder Cluster C personality disorder Unspecified personality disorder Post-traumatic stress disorder Dementia Somatoform disorder	29 (60%) 5 (10%) 6 (13%) 5 (10%) 9 (19%) 8 (17%) 3 (6%) 8 (17%) 3 (6%) 2 (4%) 1 (2%) 1 (2%) 1 (2%)
Substance Use Disorder	
No Yes Unknown	32 (67%) 14 (29%) 2 (4%)
Previous Self-harm or Suicide Attempt	
No Within 3 months prior to death More than 3 months prior to death Yes, timeline unknown Unknown	21 (44%) 12 (25%) 9 (19%) 2 (4%) 4 (8%)

Overall, a wide range of diagnoses were represented in cases of suicide in elderly patients, with depression being the most common primary diagnosis (23/48; 48%) and most common diagnosis overall (29/48; 60%). A history of substance use disorder (including alcohol use disorder) was identified in 14/48 (29%) of cases, including two cases in whom the most recent substance use occurred more than 3 months prior to death and four in which the most time of recent use was unknown. Nearly half (23/48; 48%) of cases had a history of suicide attempt or self-harm, with twelve of those cases having self-harm or a suicide attempt in the 3 months prior to death.

A greater proportion of males had a history of substance use (Male: 36%; Female: 21%) and had no history of previous self-harm or suicide attempts (Male: 50%; Female 39%). A higher proportion of females had a diagnosis of depression (Male: 52%; Female 74%), whereas a higher proportion of males had a diagnosis of adjustment disorder (Male: 21%; Female 0%) or grief/bereavement (Male: 10%; Female: 0%). A similar trend was observed for primary diagnoses, in which a much greater proportion of females had a primary diagnosis of depression than males (Male: 38%; Female: 63%). A greater proportion of females had a history of suicide attempts (Male: 46%; Female: 61%), especially in the preceding 3 months (Male: 23%; Female: 33%).

## 4 Discussion

Our findings are generally consistent with previous data on suicide in the elderly and suicide in patients receiving mental health services. In particular, we observe similar patterns in gender, ethnicity, recently identified medical illness, and diagnosis, with some differences in suicide method.

Similar to previous studies, males accounted for a greater proportion of suicides compared to females, though in our study a smaller proportion of cases were male (60% male) when compared to previous studies of suicide in older adults that reported proportions of 77% (7), 83.% (4), and 73% (6). This may be due to the decreased likelihood of men to seek help for mental health difficulties (8), leading to a lower proportion of men dying from suicide having previous contact with mental health services. These findings also suggest that a greater proportion of suicides in older women occur in the context of contact with mental health services, which is consistent with previous general population data in the U.S (5). Consequently, efforts to improve suicide prevention for older women in the mental healthcare system may be especially effective in reducing suicide in this group.

Cases of suicide identified were predominantly Caucasian, which is similar to U.S. data on suicide in older adults from the National Violent Death Reporting System (4). Of note, while Indigenous populations are a group at particularly elevated risk of suicide in Canada (9), no cases in this study were identified as Indigenous. This may be due in part to mental health services’ colonial roots, which negatively impact the willingness of Indigenous populations to engage with the system (9). It is also possible that patients may have been reluctant to identify as Indigenous, due to potential concerns on how this may impact the way they are treated. This in turn would lead to underrepresentation of Indigenous populations in data on suicide within mental health services.

Similar to other studies of suicide in older adults (4, 6), hanging was identified as one of the most common suicide methods. In this study, overdose was tied with hanging as the most common suicide method, which is similar to U.S. data (4) but differs from findings in a study in Italy (6). Both of the aforementioned studies identified firearms as a common suicide method overall, and more commonly used by men. In contrast, we only identified two cases of suicide in which firearms were used, though both cases occurred in men. This difference may be due to the difference in culture around firearms, especially in the U.S. where firearms were the most commonly used method (4). Furthermore, suicide risk assessment within mental healthcare settings typically involves asking about access to means. If a patient receiving mental health services is identified as at risk of suicide and has access to a firearm, steps may have been taken to limit this access at least in the interim period of elevated risk. This hypothesis is supported by a comparison of our findings to general population data from Statistics Canada, in which firearms were the third most commonly used method (16%) (10). The relatively smaller proportion of suicides arising from firearms in mental healthcare settings supports the importance of assessing for and limiting access to suicide means, especially firearms.

Our findings on suicide methods are similar to Statistics Canada data for all age groups, with hanging being the most common method in men and overdose being the most common method in women (10). Furthermore, we note that all suicides occurring in inpatient settings in our study occurred via hanging. As hanging has been identified as the most common method of suicide in inpatient settings in other studies (11, 12), our findings further demonstrate the importance of limiting access to potential ligatures and fixture points in inpatient settings. As three-quarters of suicide deaths among admitted/recently discharged patients occurred while the patient was on pass or recently discharged, our findings also indicate the importance of assessing suicide risk prior to patients leaving on passes and prior to discharge.

The presence of a non-psychiatric medical issue was identified in more than two-thirds of cases, with newly identified medical issues being the most common preceding adverse event. This is consistent with data on suicides in older adults in the community (4, 6), though we note that in our data newly identified medical illness was still only present in 22% of all cases, suggesting that, while it may often be a factor contributing to overall suicide risk, it is the major precipitant in only about one quarter of suicides. Previous data on suicides noted non-psychiatric health concerns as being more predominant in older men and mental health concerns as being more predominant in older women, and this was also suggested by our data, underscoring the importance of addressing mental health in older adults, especially men.

Depression was the most commonly identified diagnosis among all suicides, though older women were much more likely to have a diagnosis of depression than older men (both among all diagnoses and as primary diagnosis). Conversely, no older women had a diagnosis of adjustment disorder or grief/bereavement, though both diagnoses were present in cases of suicide in older men. Similar to men’s lower engagement with mental health services noted above, it is possible that this reflects a difference in the manner that men and women engage with the mental healthcare system and/or the manner in which the mental healthcare system engages with male and female patients. It’s possible that men are more likely to minimize the breadth, impact, or chronicity of mental health symptoms and/or that providers are more likely to underestimate the severity of symptoms that older men experience. Notably, a history of suicide attempts was much more common in suicides in older women compared to older men, with one-third having a suicide attempt in the preceding three months. This finding underlines the importance of assessing and accounting for recent suicide attempts especially in older women with depression. Previous studies examining suicide in older women suggest that physical activity and behavioral activation or other psychotherapeutic interventions may have been more effective than pharmacotherapy in reducing suicidality in this population (5), suggesting that it is essential that these treatments are readily available to this population in mental healthcare settings.

## 5 Conclusion

Our findings identify factors in cases of suicide in older adults receiving mental health services that are similar to those described in suicides in older adults in the general population and in patients with psychiatric hospitalizations. We identified a greater number of suicides in older males compared to older females, with the majority of cases being Caucasian. Hanging and overdose were the most common suicide methods used. Suicide involving firearms was less common when compared to general population data, potentially due to the routine assessment and restriction of access to means that occurs in mental healthcare treatment. The presence of non-psychiatric medical illness is common in cases of suicide in older adults and newly diagnosed medical illness is the most common adverse event occurring prior to suicide in this population. Depression was diagnosed less frequently in cases of suicide in older men when compared to suicide in older women, with more diagnoses of adjustment disorder or bereavement in cases of suicide in older men. A previous suicide attempt was more common in suicides in older women than in older men.

Our data supports many current best practices in suicide prevention, including minimizing ligatures and anchor points in inpatient psychiatric settings, assessing for and restricting access to means in at-risk patients receiving psychiatric assessment, and assessing suicide risk in hospitalized patients prior to passes and discharge.

## Data availability statement

The datasets presented in this article are not readily available because they include data (such as age at time of death) that could be used to identify specific individuals, as well as details around individual circumstances of death. Requests to access the datasets should be directed to eric.chan1@ucalgary.ca.

## Ethics statement

The studies involving humans were approved by Conjoint Health Research Ethics Board, University of Calgary (REB23-1089). The studies were conducted in accordance with the local legislation and institutional requirements. Written informed consent for participation was not required from the participants or the participants’ legal guardians/next of kin in accordance with the national legislation and institutional requirements.

## Author contributions

EC: Writing – review & editing, Writing – original draft, Project administration, Methodology, Investigation, Formal Analysis, Conceptualization. KC: Writing – review & editing, Investigation, Data curation. LG: Writing – review & editing, Supervision, Investigation.
