# Supplementary material for: Risk factors and methods in suicides of elderly patients connected to mental health services from 1999–2024
Source: Front Psychiatry. 2024 Jun 17;15:1425371. doi: 10.3389/fpsyt.2024.1425371 (PMC11215130; doi:10.3389/fpsyt.2024.1425371)
Supplement: Supplementary file 1 [file DataSheet_1.pdf]

Crosstab

			AgeCat		
			60-69	70+	Total
Gender	Female	Count	14	5	19
		% within Gender	73.7%	26.3%	100.0%
		% within AgeCat	41.2%	35.7%	39.6%
		% of Total	29.2%	10.4%	39.6%
	Male	Count	20	9	29
		% within Gender	69.0%	31.0%	100.0%
		% within AgeCat	58.8%	64.3%	60.4%
		% of Total	41.7%	18.8%	60.4%
Total		Count	34	14	48
		% within Gender	70.8%	29.2%	100.0%
		% within AgeCat	100.0%	100.0%	100.0%
		% of Total	70.8%	29.2%	100.0%

Chi-Square Tests

	Value	df	Asymptotic Significance (2-sided)	Exact Sig. (2-sided)	Exact Sig. (1-sided)	Point Probability
Pearson Chi-Square	.124[a]	1	.725	.759	.493	
Continuity Correction[b]	.001	1	.978			
Likelihood Ratio	.125	1	.724	.759	.493	
Fisher's Exact Test				1.000	.493	
Linear-by-Linear Association	.121[c]	1	.728	.759	.493	.241
N of Valid Cases	48					

a. 0 cells (.0%) have expected count less than 5. The minimum expected count is 5.54.

b. Computed only for a 2x2 table

c. The standardized statistic is .348.

Crosstab

			Marital Status					Total
			Married	Single	Divorced	Separated	Widowed	
Gender	Female	Count	11	2	5	0	1	19
		% within Gender	57.9%	10.5%	26.3%	0.0%	5.3%	100.0%
		% within Marital Status	50.0%	28.6%	50.0%	0.0%	16.7%	39.6%
		% of Total	22.9%	4.2%	10.4%	0.0%	2.1%	39.6%
	Male	Count	11	5	5	3	5	29
		% within Gender	37.9%	17.2%	17.2%	10.3%	17.2%	100.0%
		% within Marital Status	50.0%	71.4%	50.0%	100.0%	83.3%	60.4%
		% of Total	22.9%	10.4%	10.4%	6.3%	10.4%	60.4%
Total		Count	22	7	10	3	6	48
		% within Gender	45.8%	14.6%	20.8%	6.3%	12.5%	100.0%
		% within Marital Status	100.0%	100.0%	100.0%	100.0%	100.0%	100.0%
		% of Total	45.8%	14.6%	20.8%	6.3%	12.5%	100.0%

Chi-Square Tests

	Value	df	Asymptotic Significance (2-sided)	Exact Sig. (2-sided)
Pearson Chi-Square	5.090[a]	4	.278	.301
Likelihood Ratio	6.300	4	.178	.253
Fisher-Freeman-Halton Exact Test	4.502			.341
N of Valid Cases	48			

a. 7 cells (70.0%) have expected count less than 5. The minimum expected count is 1.19.

Crosstab

			Employment status							
			employed	partially employed - disability income/AISH	retired	unemployed	unemployed - disabiltiy income/AISH	unknown	yes	Total
Gender	Female	Count	0	1	9	6	2	1	0	19
		% within Gender	0.0%	5.3%	47.4%	31.6%	10.5%	5.3%	0.0%	100.0%
		% within Employment status	0.0%	100.0%	37.5%	46.2%	50.0%	33.3%	0.0%	39.6%
		% of Total	0.0%	2.1%	18.8%	12.5%	4.2%	2.1%	0.0%	39.6%
	Male	Count	1	0	15	7	2	2	2	29
		% within Gender	3.4%	0.0%	51.7%	24.1%	6.9%	6.9%	6.9%	100.0%
		% within Employment status	100.0%	0.0%	62.5%	53.8%	50.0%	66.7%	100.0%	60.4%
		% of Total	2.1%	0.0%	31.3%	14.6%	4.2%	4.2%	4.2%	60.4%
Total		Count	1	1	24	13	4	3	2	48
		% within Gender	2.1%	2.1%	50.0%	27.1%	8.3%	6.3%	4.2%	100.0%
		% within Employment status	100.0%	100.0%	100.0%	100.0%	100.0%	100.0%	100.0%	100.0%
		% of Total	2.1%	2.1%	50.0%	27.1%	8.3%	6.3%	4.2%	100.0%

Chi-Square Tests

	Value	df	Asymptotic Significance (2-sided)	Exact Sig. (2-sided)
Pearson Chi-Square	4.001[a]	6	.677	.807
Likelihood Ratio	5.379	6	.496	.774
Fisher-Freeman-Halton Exact Test	3.817			.842
N of Valid Cases	48			

a. 10 cells (71.4%) have expected count less than 5. The minimum expected count is .40.

Crosstab

			Method									Total
			Unknown	Laceration	Drowning	Overdose	Gunshot	Hanging	Carbon Monoxide	Fall from Height	Asphyxiation	
Gender	Female	Count	3	0	1	8	0	5	1	0	1	19
		% within Gender	15.8%	0.0%	5.3%	42.1%	0.0%	26.3%	5.3%	0.0%	5.3%	100.0%
		% within Method	42.9%	0.0%	25.0%	53.3%	0.0%	33.3%	100.0%	0.0%	100.0%	39.6%
		% of Total	6.3%	0.0%	2.1%	16.7%	0.0%	10.4%	2.1%	0.0%	2.1%	39.6%
	Male	Count	4	1	3	7	2	10	0	2	0	29
		% within Gender	13.8%	3.4%	10.3%	24.1%	6.9%	34.5%	0.0%	6.9%	0.0%	100.0%
		% within Method	57.1%	100.0%	75.0%	46.7%	100.0%	66.7%	0.0%	100.0%	0.0%	60.4%
		% of Total	8.3%	2.1%	6.3%	14.6%	4.2%	20.8%	0.0%	4.2%	0.0%	60.4%
Total		Count	7	1	4	15	2	15	1	2	1	48
		% within Gender	14.6%	2.1%	8.3%	31.3%	4.2%	31.3%	2.1%	4.2%	2.1%	100.0%
		% within Method	100.0%	100.0%	100.0%	100.0%	100.0%	100.0%	100.0%	100.0%	100.0%	100.0%
		% of Total	14.6%	2.1%	8.3%	31.3%	4.2%	31.3%	2.1%	4.2%	2.1%	100.0%

Chi-Square Tests

	Value	df	Asymptotic Significance (2-sided)	Exact Sig. (2-sided)
Pearson Chi-Square	8.146[a]	8	.419	.458
Likelihood Ratio	10.561	8	.228	.429
Fisher-Freeman-Halton Exact Test	7.196			.559
N of Valid Cases	48			

a. 14 cells (77.8%) have expected count less than 5. The minimum expected count is .40.

Crosstab

			Losses/ adverse life events within last 6 months				Total
				No	Yes	Unknown	
Gender	Female	Count	0	6	13	0	19
		% within Gender	0.0%	31.6%	68.4%	0.0%	100.0%
		% within Losses/ adverse life events within last 6 months	0.0%	46.2%	40.6%	0.0%	39.6%
		% of Total	0.0%	12.5%	27.1%	0.0%	39.6%
	Male	Count	1	7	19	2	29
		% within Gender	3.4%	24.1%	65.5%	6.9%	100.0%
		% within Losses/ adverse life events within last 6 months	100.0%	53.8%	59.4%	100.0%	60.4%
		% of Total	2.1%	14.6%	39.6%	4.2%	60.4%
Total		Count	1	13	32	2	48
		% within Gender	2.1%	27.1%	66.7%	4.2%	100.0%
		% within Losses/ adverse life events within last 6 months	100.0%	100.0%	100.0%	100.0%	100.0%
		% of Total	2.1%	27.1%	66.7%	4.2%	100.0%

Chi-Square Tests

	Value	df	Asymptotic Significance (2-sided)	Exact Sig. (2-sided)
Pearson Chi-Square	2.215[a]	3	.529	.651
Likelihood Ratio	3.269	3	.352	.574
Fisher-Freeman-Halton Exact Test	1.852			.744
N of Valid Cases	48			

a. 4 cells (50.0%) have expected count less than 5. The minimum expected count is .40.

Crosstab

			NewMedicalIssue		
			No	Yes	Total
Gender	Female	Count	15	4	19
		% within Gender	78.9%	21.1%	100.0%
		% within NewMedicalIssue	39.5%	40.0%	39.6%
		% of Total	31.3%	8.3%	39.6%
	Male	Count	23	6	29
		% within Gender	79.3%	20.7%	100.0%
		% within NewMedicalIssue	60.5%	60.0%	60.4%
		% of Total	47.9%	12.5%	60.4%
Total		Count	38	10	48
		% within Gender	79.2%	20.8%	100.0%
		% within NewMedicalIssue	100.0%	100.0%	100.0%
		% of Total	79.2%	20.8%	100.0%

Chi-Square Tests

	Value	df	Asymptotic Significance (2-sided)	Exact Sig. (2-sided)	Exact Sig. (1-sided)	Point Probability
Pearson Chi-Square	.001[a]	1	.976	1.000	.624	
Continuity Correction[b]	.000	1	1.000			
Likelihood Ratio	.001	1	.976	1.000	.624	
Fisher's Exact Test				1.000	.624	
Linear-by-Linear Association	.001[c]	1	.976	1.000	.624	.281
N of Valid Cases	48					

a. 1 cells (25.0%) have expected count less than 5. The minimum expected count is 3.96.

b. Computed only for a 2x2 table

c. The standardized statistic is -.030.

Crosstab

			IllnessDeathLovedOne		
			No	Yes	Total
Gender	Female	Count	17	2	19
		% within Gender	89.5%	10.5%	100.0%
		% within IllnessDeathLovedOne	40.5%	33.3%	39.6%
		% of Total	35.4%	4.2%	39.6%
	Male	Count	25	4	29
		% within Gender	86.2%	13.8%	100.0%
		% within IllnessDeathLovedOne	59.5%	66.7%	60.4%
		% of Total	52.1%	8.3%	60.4%
Total		Count	42	6	48
		% within Gender	87.5%	12.5%	100.0%
		% within IllnessDeathLovedOne	100.0%	100.0%	100.0%
		% of Total	87.5%	12.5%	100.0%

Chi-Square Tests

	Value	df	Asymptotic Significance (2-sided)	Exact Sig. (2-sided)	Exact Sig. (1-sided)	Point Probability
Pearson Chi-Square	.112[a]	1	.738	1.000	.554	
Continuity Correction[b]	.000	1	1.000			
Likelihood Ratio	.114	1	.736	1.000	.554	
Fisher's Exact Test				1.000	.554	
Linear-by-Linear Association	.110[c]	1	.741	1.000	.554	.331
N of Valid Cases	48					

a. 2 cells (50.0%) have expected count less than 5. The minimum expected count is 2.38.

b. Computed only for a 2x2 table

c. The standardized statistic is .331.

Crosstab

			LossofSocialSupport No	LossofSocialSupport Yes	Total
Gender	Female	Count	17	2	19
		% within Gender	89.5%	10.5%	100.0%
		% within LossofSocialSupport	39.5%	40.0%	39.6%
		% of Total	35.4%	4.2%	39.6%
	Male	Count	26	3	29
		% within Gender	89.7%	10.3%	100.0%
		% within LossofSocialSupport	60.5%	60.0%	60.4%
		% of Total	54.2%	6.3%	60.4%
Total		Count	43	5	48
		% within Gender	89.6%	10.4%	100.0%
		% within LossofSocialSupport	100.0%	100.0%	100.0%
		% of Total	89.6%	10.4%	100.0%

Chi-Square Tests

	Value	df	Asymptotic Significance (2-sided)	Exact Sig. (2-sided)	Exact Sig. (1-sided)	Point Probability
Pearson Chi-Square	.000[a]	1	.984	1.000	.667	
Continuity Correction[b]	.000	1	1.000			
Likelihood Ratio	.000	1	.984	1.000	.667	
Fisher's Exact Test				1.000	.667	
Linear-by-Linear Association	.000[c]	1	.984	1.000	.667	.365
N of Valid Cases	48					

a. 2 cells (50.0%) have expected count less than 5. The minimum expected count is 1.98.

b. Computed only for a 2x2 table

c. The standardized statistic is -.020.

Crosstab

			LossofHousing		
			No	Yes	Total
Gender	Female	Count	17	2	19
		% within Gender	89.5%	10.5%	100.0%
		% within LossofHousing	40.5%	33.3%	39.6%
		% of Total	35.4%	4.2%	39.6%
	Male	Count	25	4	29
		% within Gender	86.2%	13.8%	100.0%
		% within LossofHousing	59.5%	66.7%	60.4%
		% of Total	52.1%	8.3%	60.4%
Total		Count	42	6	48
		% within Gender	87.5%	12.5%	100.0%
		% within LossofHousing	100.0%	100.0%	100.0%
		% of Total	87.5%	12.5%	100.0%

Chi-Square Tests

	Value	df	Asymptotic Significance (2-sided)	Exact Sig. (2-sided)	Exact Sig. (1-sided)	Point Probability
Pearson Chi-Square	.112[a]	1	.738	1.000	.554	
Continuity Correction[b]	.000	1	1.000			
Likelihood Ratio	.114	1	.736	1.000	.554	
Fisher's Exact Test				1.000	.554	
Linear-by-Linear Association	.110[c]	1	.741	1.000	.554	.331
N of Valid Cases	48					

a. 2 cells (50.0%) have expected count less than 5. The minimum expected count is 2.38.

b. Computed only for a 2x2 table

c. The standardized statistic is .331.

Crosstab

			FinancialIssues No	FinancialIssues Yes	Total
Gender	Female	Count	18	1	19
		% within Gender	94.7%	5.3%	100.0%
		% within FinancialIssues	40.0%	33.3%	39.6%
		% of Total	37.5%	2.1%	39.6%
	Male	Count	27	2	29
		% within Gender	93.1%	6.9%	100.0%
		% within FinancialIssues	60.0%	66.7%	60.4%
		% of Total	56.3%	4.2%	60.4%
Total		Count	45	3	48
		% within Gender	93.8%	6.3%	100.0%
		% within FinancialIssues	100.0%	100.0%	100.0%
		% of Total	93.8%	6.3%	100.0%

Chi-Square Tests

	Value	df	Asymptotic Significance (2-sided)	Exact Sig. (2-sided)	Exact Sig. (1-sided)	Point Probability
Pearson Chi-Square	.052[a]	1	.819	1.000	.657	
Continuity Correction[b]	.000	1	1.000			
Likelihood Ratio	.053	1	.817	1.000	.657	
Fisher's Exact Test				1.000	.657	
Linear-by-Linear Association	.051[c]	1	.821	1.000	.657	.446
N of Valid Cases	48					

a. 2 cells (50.0%) have expected count less than 5. The minimum expected count is 1.19.

b. Computed only for a 2x2 table

c. The standardized statistic is .226.

Crosstab

			SocialConflict		Total
			No	Yes	
Gender	Female	Count	18	1	19
		% within Gender	94.7%	5.3%	100.0%
		% within SocialConflict	38.3%	100.0%	39.6%
		% of Total	37.5%	2.1%	39.6%
	Male	Count	29	0	29
		% within Gender	100.0%	0.0%	100.0%
		% within SocialConflict	61.7%	0.0%	60.4%
		% of Total	60.4%	0.0%	60.4%
Total		Count	47	1	48
		% within Gender	97.9%	2.1%	100.0%
		% within SocialConflict	100.0%	100.0%	100.0%
		% of Total	97.9%	2.1%	100.0%

Chi-Square Tests

	Value	df	Asymptotic Significance (2-sided)	Exact Sig. (2-sided)	Exact Sig. (1-sided)	Point Probability
Pearson Chi-Square	1.559[a]	1	.212	.396	.396	
Continuity Correction[b]	.046	1	.830			
Likelihood Ratio	1.886	1	.170	.396	.396	
Fisher's Exact Test				.396	.396	
Linear-by-Linear Association	1.526[c]	1	.217	.396	.396	.396
N of Valid Cases	48					

a. 2 cells (50.0%) have expected count less than 5. The minimum expected count is .40.

b. Computed only for a 2x2 table

c. The standardized statistic is -1.235.

Crosstab

			Depression		
			No	Yes	Total
Gender	Female	Count	5	14	19
		% within Gender	26.3%	73.7%	100.0%
		% within Depression	26.3%	48.3%	39.6%
		% of Total	10.4%	29.2%	39.6%
	Male	Count	14	15	29
		% within Gender	48.3%	51.7%	100.0%
		% within Depression	73.7%	51.7%	60.4%
		% of Total	29.2%	31.3%	60.4%
Total		Count	19	29	48
		% within Gender	39.6%	60.4%	100.0%
		% within Depression	100.0%	100.0%	100.0%
		% of Total	39.6%	60.4%	100.0%

Chi-Square Tests

	Value	df	Asymptotic Significance (2-sided)	Exact Sig. (2-sided)	Exact Sig. (1-sided)	Point Probability
Pearson Chi-Square	2.315[a]	1	.128	.147	.111	
Continuity Correction[b]	1.488	1	.223			
Likelihood Ratio	2.375	1	.123	.147	.111	
Fisher's Exact Test				.147	.111	
Linear-by-Linear Association	2.267[c]	1	.132	.147	.111	.078
N of Valid Cases	48					

a. 0 cells (.0%) have expected count less than 5. The minimum expected count is 7.52.

b. Computed only for a 2x2 table

c. The standardized statistic is -1.506.

Crosstab

			Primary Diagnosis Recoded										
			Depression	Bipolar disorder	Adjustment disorder	Schizophrenia or other psychotic disorder	Anxiety	Substance-related disorder	Grief	Cluster B personality disorder	Dementia	Somatoform disorder	Total
Gender	Female	Count	12	0	0	2	3	1	0	1	0	0	19
		% within Gender	63.2%	0.0%	0.0%	10.5%	15.8%	5.3%	0.0%	5.3%	0.0%	0.0%	100.0%
		% within Primary Diagnosis Recoded	52.2%	0.0%	0.0%	50.0%	60.0%	20.0%	0.0%	100.0%	0.0%	0.0%	39.6%
		% of Total	25.0%	0.0%	0.0%	4.2%	6.3%	2.1%	0.0%	2.1%	0.0%	0.0%	39.6%
	Male	Count	11	3	3	2	2	4	2	0	1	1	29
		% within Gender	37.9%	10.3%	10.3%	6.9%	6.9%	13.8%	6.9%	0.0%	3.4%	3.4%	100.0%
		% within Primary Diagnosis Recoded	47.8%	100.0%	100.0%	50.0%	40.0%	80.0%	100.0%	0.0%	100.0%	100.0%	60.4%
		% of Total	22.9%	6.3%	6.3%	4.2%	4.2%	8.3%	4.2%	0.0%	2.1%	2.1%	60.4%
Total		Count	23	3	3	4	5	5	2	1	1	1	48
		% within Gender	47.9%	6.3%	6.3%	8.3%	10.4%	10.4%	4.2%	2.1%	2.1%	2.1%	100.0%
		% within Primary Diagnosis Recoded	100.0%	100.0%	100.0%	100.0%	100.0%	100.0%	100.0%	100.0%	100.0%	100.0%	100.0%
		% of Total	47.9%	6.3%	6.3%	8.3%	10.4%	10.4%	4.2%	2.1%	2.1%	2.1%	100.0%

Chi-Square Tests

	Value	df	Asymptotic Significance (2-sided)	Exact Sig. (2-sided)	Exact Sig. (1-sided)	Point Probability
Pearson Chi-Square	11.457[a]	9	.246	.214		
Likelihood Ratio	15.323	9	.082	.178		
Fisher-Freeman-Halton Exact Test	10.087			.272		
Linear-by-Linear Association	1.444[b]	1	.230	.251	.128	.023
N of Valid Cases	48					

a. 18 cells (90.0%) have expected count less than 5. The minimum expected count is .40.

b. The standardized statistic is 1.202.

Crosstab

			SubstanceUseYesNo			
			No	Yes	Unknown	Total
Gender	Female	Count	14	4	1	19
		% within Gender	73.7%	21.1%	5.3%	100.0%
		% within SubstanceUseYesNo	43.8%	28.6%	100.0%	40.4%
		% of Total	29.8%	8.5%	2.1%	40.4%
	Male	Count	18	10	0	28
		% within Gender	64.3%	35.7%	0.0%	100.0%
		% within SubstanceUseYesNo	56.3%	71.4%	0.0%	59.6%
		% of Total	38.3%	21.3%	0.0%	59.6%
Total		Count	32	14	1	47
		% within Gender	68.1%	29.8%	2.1%	100.0%
		% within SubstanceUseYesNo	100.0%	100.0%	100.0%	100.0%
		% of Total	68.1%	29.8%	2.1%	100.0%

Chi-Square Tests

	Value	df	Asymptotic Significance (2-sided)	Exact Sig. (2-sided)	Exact Sig. (1-sided)	Point Probability
Pearson Chi-Square	2.437[a]	2	.296	.326		
Likelihood Ratio	2.810	2	.245	.326		
Fisher-Freeman-Halton Exact Test	2.303			.326		
Linear-by-Linear Association	.071[b]	1	.790	1.000	.512	.217
N of Valid Cases	47					

a. 2 cells (33.3%) have expected count less than 5. The minimum expected count is .40.

b. The standardized statistic is .266.

Crosstab

			PreviousSelfharmorAttempts					
			No	Within 3 months	More than 3 months prior	Yes, timeline unknown	Unknown	Total
Gender	Female	Count	7	6	4	1	0	18
		% within Gender	38.9%	33.3%	22.2%	5.6%	0.0%	100.0%
		% within PreviousSelfharmorAttempts	33.3%	50.0%	44.4%	50.0%	0.0%	39.1%
		% of Total	15.2%	13.0%	8.7%	2.2%	0.0%	39.1%
	Male	Count	14	6	5	1	2	28
		% within Gender	50.0%	21.4%	17.9%	3.6%	7.1%	100.0%
		% within PreviousSelfharmorAttempts	66.7%	50.0%	55.6%	50.0%	100.0%	60.9%
		% of Total	30.4%	13.0%	10.9%	2.2%	4.3%	60.9%
Total		Count	21	12	9	2	2	46
		% within Gender	45.7%	26.1%	19.6%	4.3%	4.3%	100.0%
		% within PreviousSelfharmorAttempts	100.0%	100.0%	100.0%	100.0%	100.0%	100.0%
		% of Total	45.7%	26.1%	19.6%	4.3%	4.3%	100.0%

Chi-Square Tests

	Value	df	Asymptotic Significance (2-sided)	Exact Sig. (2-sided)	Exact Sig. (1-sided)	Point Probability
Pearson Chi-Square	2.383[a]	4	.666	.704		
Likelihood Ratio	3.071	4	.546	.704		
Fisher-Freeman-Halton Exact Test	2.394			.746		
Linear-by-Linear Association	.003[b]	1	.953	1.000	.535	.106
N of Valid Cases	46					

a. 6 cells (60.0%) have expected count less than 5. The minimum expected count is .78.

b. The standardized statistic is .059.
